# Toxicological Effects of Leachates Extracted from Photocatalytic Concrete Blocks with Nano-TiO_2_ on *Daphnia magna*

**DOI:** 10.3390/nano14171447

**Published:** 2024-09-04

**Authors:** Fernanda Facin, João Victor Staub de Melo, Rodrigo Costa Puerari, William Gerson Matias

**Affiliations:** 1 Department of Civil Engineering, Federal University of Santa Catarina (UFSC), Rua Engenheiro Agronômico Andrei Cristian Ferreira, Trindade, Florianópolis 88040-900, SC, Brazil; fernanda.facin@posgrad.ufsc.br; 2 Department of Sanitary and Environmental Engineering, Federal University of Santa Catarina (UFSC), Rua Engenheiro Agronômico Andrei Cristian Ferreira, Trindade, Florianópolis 88040-900, SC, Brazil; rodrigo.puerari@posgrad.ufsc.br (R.C.P.); william.g.matias@ufsc.br (W.G.M.)

**Keywords:** nanoparticles, titanium dioxide, cement, paving, leaching, toxicological evaluation

## Abstract

The incorporation of titanium dioxide nanoparticles into concrete blocks for paving adds photocatalytic functionality to the cementitious matrix, providing self-cleaning and pollutant-degrading properties. However, wear and leaching from these pavements can release potentially toxic compounds into water bodies, affecting aquatic organisms. In this context, this study evaluated the toxicological effects of leachates from photocatalytic concrete containing nano-TiO_2_ with an average size of 10 nm and anatase crystallinity on *Daphnia magna*. Acute and chronic toxicity tests on neonates were conducted with two leachate extracts: one from reference concrete and one from photocatalytic concrete (with 9% nano-TiO_2_ added by mass of cement). In terms of acute toxicity, the reference concrete extract had an EC_50_ of 104.0 mL/L at 48 h, whereas the concrete with TiO_2_ had an EC_50_ of 64.6 mL/L at 48 h. For chronic toxicity, the leachate from reference concrete had a significant effect (*p* < 0.05) on the size parameter with an LOEC of 4 mL/L, whereas the leachate from concrete with 9% nano-TiO_2_ did not have significant toxicological effects on any of the analyzed parameters (longevity, size, reproduction, and age of first posture) (LOEC > 6.5 mL/L). Furthermore, FTIR analysis indicated that TiO_2_ nanoparticles were not detected in the leachates, suggesting efficient anchoring within the cementitious matrix. The results indicate that there was no increase in the chronic toxicity of the leachate from the cementitious matrix when nanoparticles were added at a 9% mass ratio of cement.

## 1. Introduction

The incorporation of titanium dioxide nanoparticles (nano-TiO_2_) in concrete blocks for pavements has been extensively studied and documented in the literature [1,2], as it adds photocatalytic functionality to the cementitious matrix, ensuring self-cleaning and decontaminating properties of the material [3,4]. Heterogeneous photocatalysis is a process activated by sunlight, allowing the photocatalytic process to degrade organic and inorganic pollutants present on the surface or in the surrounding atmosphere, promoting the breakdown of harmful compounds such as nitrogen oxides (NOx) and volatile organic compounds (VOCs) [5,6]. In this context, studies [7,8,9] have demonstrated that the incorporation of nano-TiO_2_ in concrete can significantly reduce the concentration of atmospheric pollutants in urban areas, contributing to improved air quality. Additionally, the self-cleaning property of photocatalytic blocks minimizes the need for maintenance and cleaning of paved surfaces, resulting in greater durability and resource savings [10]. The use of these photocatalytic concrete blocks in pavements has also been correlated with environmental sustainability, and it aligns with sustainable urban development policies and provides significant benefits for public health and the environment [11].

Despite the benefits of photocatalytic concrete, the surface of pavements in service is subject to wear and leaching [2]. Mechanical wear results from the continuous action of vehicle and pedestrian traffic [12], whereas leaching occurs due to the action of atmospheric agents and rainwater, which dissolve and carry the compounds present in the concrete pavements to the surroundings, such as water bodies (river sections, streams, natural or artificial reservoirs, lakes, or underground aquifers) [13].

In this context, studies have demonstrated that nanoparticles can exert significant toxic effects in aquatic environments, negatively impacting organisms such as *D. magna*, which underscores the need for rigorous toxicological assessments to mitigate environmental risks [14,15]. In particular, the toxicity of nanoparticles, such as titanium dioxide nanoparticles, is influenced by various factors, including size, shape, surface properties, and the chemistry of the aquatic medium [16,17]. Furthermore, the interaction of nano-TiO_2_ with the cementitious matrix can enhance its toxic effects, producing more complex leachates and presenting additional challenges for environmental risk assessments.

This study aimed to evaluate the toxicological effects of leachates extracted from photocatalytic concrete blocks with nano-TiO_2_ on the freshwater organism *D. magna*.

## 2. Materials

To achieve the proposed objective of the study, the materials used were as follows: nanoparticles, cement, and mineral aggregates.

### 2.1. Nanoparticles

Nanometric titanium dioxide (nano-TiO_2_) was selected as the semiconductor for this investigation. The use of these nanoparticles is justified by their widespread application in cementitious matrices for heterogeneous photocatalysis, as documented in the literature [1,2,3,4,8,9,10]. Table 1 presents the chemical and physical characteristics of the nanometric powder. The data were provided by the manufacturer (Nanostructured & Amorphous Materials, Inc., Houston, TX, USA) [18].

**Figure 1:**
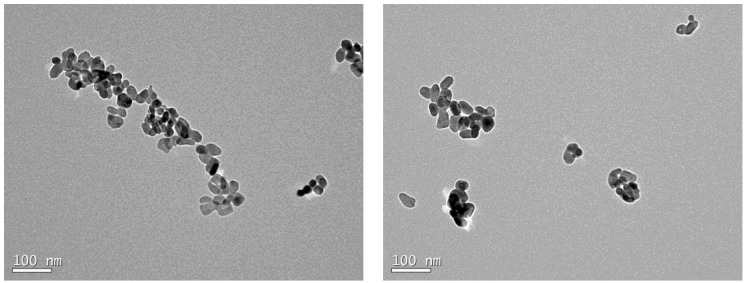
Micrographs of TiO_2_ nanoparticles.

To confirm the nanometric sizes of the particles, the powder was examined via transmission electron microscopy (TEM) with a JEOL instrument (model JEM-1011 TEM—100 kV equipment, Tokyo, Japan). For the application of this technique, TiO_2_ particles were suspended in ultrapure water at a concentration of 1 g/L and dispersed via ultrasonication (Qsonica Q 500 W, Newtown, CT, USA) for 2 min at 115 W. Subsequently, the solution was dropped onto a grid (carbon film coated with 300 mesh, Cu) and stored in a vacuum desiccator for 24 h to dry. After the 24 h period, the micrographs presented in Figure 1 were obtained, confirming the nanometric sizes of the particles.

In addition to the characteristics described by the manufacturer for nano-TiO_2_, further analyses were conducted to understand the behavior of the powder in a liquid medium. In this regard, the zeta potential, effective diameter, polydispersity index, and pH of the colloidal suspension of the nanoparticles in ultrapure water and the culture and/or test media for *D. magna* (ISO medium—acute toxicity and M4 medium—chronic toxicity) were determined. The suspensions were prepared at a concentration of 100 mg/L nanoparticles in the medium. All the suspensions were dispersed via ultrasonication (Qsonica Q 500 W) at a power of 115 W for 2 min. Subsequently, the pH was determined at 20 ± 1 °C (Alfakit, AT-355), and the zeta potential, effective diameter, and polydispersity index were measured in triplicate via a Nanobrook 90Plus PALS device (Brookhaven, New York, NY, USA). Table 2 presents the results of the suspension characterization.

According to Table 2, the zeta potential values measured for the nano-TiO_2_ suspensions varied depending on the medium, being 12.89 ± 0.38 mV in ultrapure water, −2.85 ± 0.09 mV in ISO medium, and 3.84 ± 0.35 mV in M4 medium. Although the literature generally [19] associates absolute values greater than ±30 mV with high colloidal stability due to electrostatic repulsion between particles, it is important to note, as discussed by Riddick [20] in the book “Control of Colloid Stability through Zeta Potential”, that the stability of a colloidal suspension does not depend solely on zeta potential. Other factors, such as van der Waals interactions and the composition of the medium, also play a crucial role. Therefore, the observed values suggest a tendency toward instability in the suspensions in ISO and M4 media, possibly due to the formation of agglomerates resulting from the interaction between dissolved salts and the surfaces of the nanoparticles, which is consistent with the analysis of zeta potential, polydispersity index, and effective diameter. The polydispersity index was less than 0.3 for all media, indicating the formation of monodisperse systems, and the mean value of the effective diameter increased significantly when comparing the nanoparticles suspended in ultrapure water with those in the other two media (ISO and M4), possibly due to the higher number of salts in the solutions.

**Figure 2:**
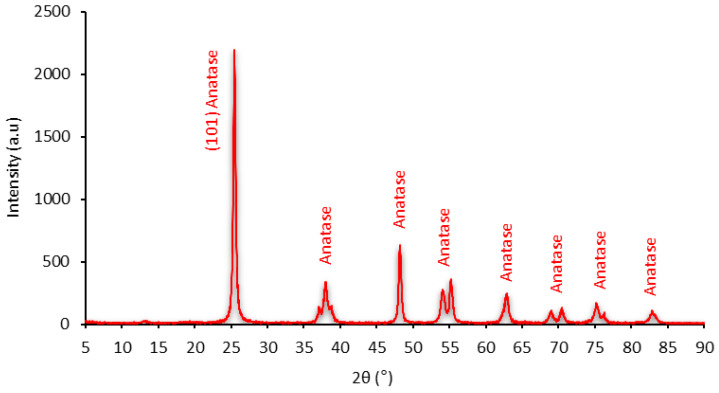
X-ray diffraction pattern of the pure nanoparticle powder.

Finally, the crystallographic structure and purity of the nano-TiO_2_ were verified via X-ray diffraction (Rigaku Miniflex II, Tokyo, Japan) and energy-dispersive X-ray spectroscopy (Shimadzu 700 HS, Tokyo, Japan), respectively. Figure 2 presents the X-ray diffraction results.

As shown in Figure 2, the X-ray diffraction (XRD) spectrum confirmed the tetragonal crystal system (anatase phase) of titanium dioxide, with the peak with the highest intensity at the (101) plane. The pattern of the results is consistent with the International Center for Diffraction Data (ICDD) card No. 21-1272 [21].

Energy-dispersive X-ray spectroscopy (EDX) was used to determine the purity of the powder, which revealed the following oxide contents: 99.463% titanium dioxide (TiO_2_), 0.34% sulfur trioxide (SO_3_), 0.104% calcium oxide (CaO), 0.049% niobium oxide (NbO), 0.027% copper oxide (CuO), and 0.017% zirconium dioxide (ZrO_2_).

### 2.2. Cement

The hydraulic binder used was Portland cement composed of pozzolan (clinker + plaster: 76–94%; limestone: 0–10%; pozzolan: 6–14%). Table 3 presents the physical and chemical characteristics of the cement. The properties were obtained in accordance with the specifications of the ABNT NBR 16697 [22] and ASTM C150/C150M [23] standards.

### 2.3. Mineral Aggregates

The mineral aggregate that was used in the concrete has a granitic mineralogical origin and has the following main properties: 8.02% powder material [24]; apparent specific mass of 2.536 kg/dm^3^ [25]; 1.43% absorbance [25]; fineness module of 3.48; and maximum diameter of 9.5 mm [25].

## 3. Methods

The methods can be summarized in the following steps: (i) production of concrete blocks for paving; (ii) leaching process of the concrete; (iii) characterization of the leachate extracts; and (iv) toxicological assessment of the leachate extracts in *D. magna*. A complete description of the procedures used in the experimental program is presented in the following subsections.

### 3.1. Production of Concrete Blocks for Paving

For this research, 6 concrete blocks (105 mm × 210 mm × 90 mm) were produced and divided into two groups: a reference group (without the addition of nanoparticles) and a photocatalytic group with nano-TiO_2_ added to the concrete at a concentration of 9% relative to the mass of Portland cement; this incorporation percentage is used in various studies on photocatalytic paving.

The base mixture used for the concrete was 1:3.5 (cement/aggregate) with a water/cement ratio of 0.427. The aggregate gradation curve followed the maximum density criterion of Füller [26], with a maximum particle diameter of 9.5 mm and a Füller–Talbot exponent of 0.45. The percentage passed through each sieve (ASTM series) was as follows: 3/8″–100%; 1/4″–83.12%; n^o^. 4–73.55%; n^o^. 8–53.84%; n^o^. 16–39.41%; n^o^. 30–28.85%; n^o^. 50–21.12%; n^o^. 100–15.46%; and n^o^. 200–11.32%.

All the concrete blocks were produced in the laboratory via a metallic mold and hydraulic press, achieving a wet specific mass of 2.339 g/cm^3^ and a compressive strength greater than 47 MPa [27] at 28 days of curing. The blocks were prepared, demolded, and cured in accordance with ASTM C192/C192M standards [28]. The photocatalytic efficiency of the blocks containing 9% nano-TiO_2_ has been thoroughly evaluated and documented in the study conducted by Rosso and Melo [2].

### 3.2. Leaching Process of the Concrete

The leaching process of the concrete was conducted according to ABNT NBR 10005 [29] based on the “Toxicity Characteristic Leaching Procedure” (TCLP) (SW-846 Method 1311; USEPA, 1994). According to the standard, test samples must have particles with a maximum size of 9.5 mm. If not, the sample must be crushed until it meets the requirements. Thus, for the leaching process, concrete particles ranging in size from 9.5 mm to 4.8 mm were extracted from the blocks. Additionally, the particles were washed in ultrapure water and allowed to dry at room temperature. This process aimed to remove the powdery material from the surface, which was generated by the extraction process.

With respect to the extraction solution used for the leaching procedure, solution 1 of the standard was used with the following formulation: 5.7 mL of glacial acetic acid, 64.3 mL of 1.0 N NaOH (sodium hydroxide), and 930 mL of ultrapure water. This solution was defined since all the samples had a pH ≤ 5 in a liquid medium (deionized water).

**Figure 3:**
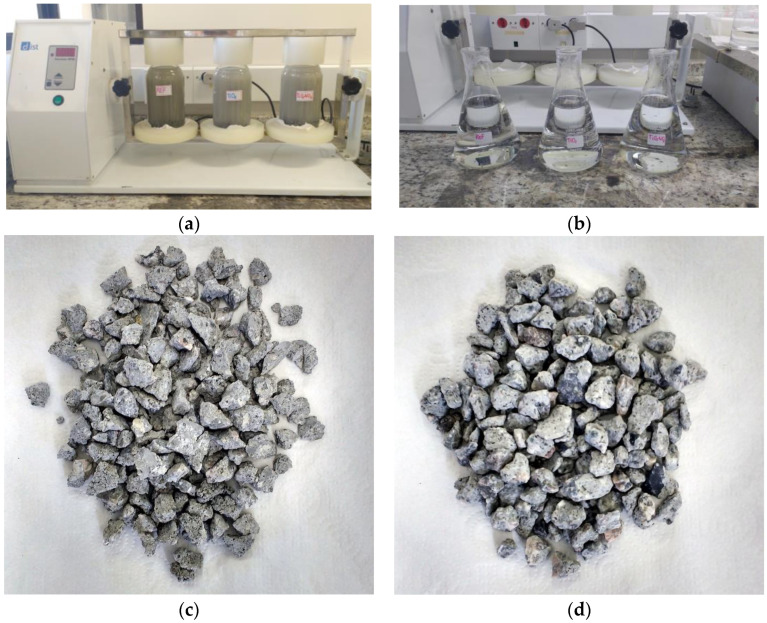
(**a**) Rotary shaker; (**b**) leachate samples after filtration; (**c**) concrete particles before leaching; and (**d**) concrete particles after leaching.

The leaching procedure was performed in a rotary flask shaker with 100 ± 0.1 g of each concrete sample (particles ranging from 9.5 mm to 4.8 mm) in flasks containing 2 L of extraction solution, in triplicates. Agitation was carried out at a speed of 30 ± 2 rpm for 18 ± 2 h at 25 °C. After agitation, the samples were filtered via a vacuum device, evaporated in an oven at 60 °C for 24 h to concentrate the leachate extract, and finally stored in a refrigerator for use in toxicological tests. Figure 3 shows the leaching procedure in the rotary shaker, the leachate extracts after filtration, and the appearance of one of the concrete samples before and after leaching.

### 3.3. Characterization of the Leachate Extracts

The characterization of the leachate extracts was conducted via two techniques: inductively coupled plasma mass spectrometry (ICP–MS) and Fourier transform infrared spectroscopy (FTIR). ICP–MS analysis (Perkin Elmer Sciex, Elan 6000, Waltham, MA, USA) was performed to quantify the presence of titanium (Ti) in the liquid samples obtained through the leaching process of the concrete. For this technique, the samples were acidified with nitric acid (HNO_3_) for the subsequent determination of the analyte of interest. FTIR analysis (Agilent Cary 660 FTIR, Santa Clara, CA, USA) was used to identify the presence of nanoparticles in the leachate extracts, specifically to determine if the particles were detached from the cementitious matrix. For FTIR analysis, the leachate samples were freeze-dried (Liotop L101, São Carlos, SP, Brazil) under vacuum at −40 °C for 24 h to obtain solid samples. Additionally, FTIR spectroscopy was also performed on the pure nano-TiO_2_ powder to facilitate the analysis of the results.

### 3.4. Toxicological Assessment of the Leachate Extracts in D. magna

For the toxicity tests, neonatal *D. magna* STRAUS, 1820 (*Cladocera*, Crustacea), aged between 2 and 26 h, obtained from females aged between 10 and 60 days, was used. The cultures followed the guidelines of ABNT NBR 12713 [30] and ISO 6341 [31] and used reconstituted water (M4 medium) with diffuse lighting, a photoperiod of 16 h of light, and a temperature of 20 ± 2 °C. The organisms were fed the species *Scenedesmus subspicatus*, now referred to as *Desmodesmus subspicatus*. It is noteworthy that weekly sensitivity tests were conducted with potassium dichromate (K_2_Cr_2_O_7_) using the organisms to ensure adherence to international standards in cultivation and to validate the toxicological tests. The culture used in the experiments was obtained and maintained at the Laboratory of Environmental Toxicology (LABTOX/UFSC).

The selection of *D. magna* neonates for the toxicological tests was based on their position at lower trophic levels. This characteristic is important for assessing the amplification of contamination along the food chain. Additionally, *D. magna* is widely used in scientific research [32,33,34,35].

Toxicity tests were performed with leachate extracts from two types of concrete: reference concrete and concrete with 9% nano-TiO_2_. Additionally, for comparative purposes, tests were also conducted with a pure TiO_2_ nanoparticle powder. Two toxicological tests were conducted with *D. magna* neonates: acute toxicity and chronic toxicity.

**Figure 4:**
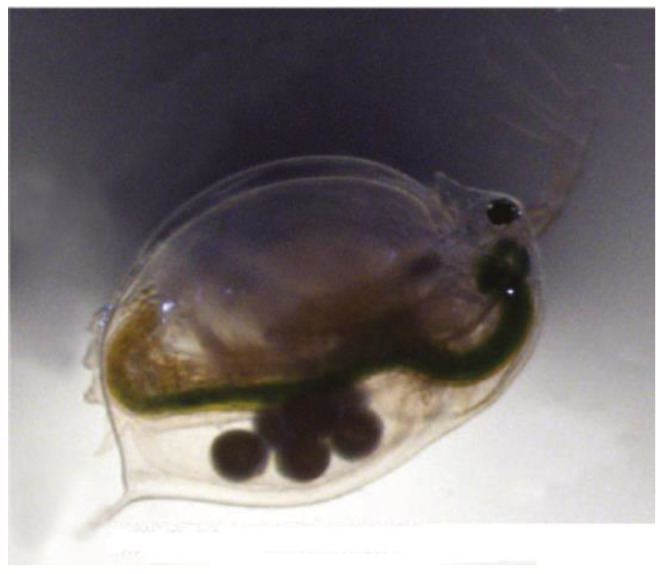
*D. magna* at the end of the chronic toxicity test, using the diluent medium (M4) without the presence of toxic agents such as nanoparticles and/or leachates.

Figure 4 shows *D. magna* at the end of the chronic toxicity test, using the diluent medium (M4) without the presence of toxic agents such as nanoparticles and/or leachates. The image provides a visual reference for the morphology, color, and size of the organisms.

#### 3.4.1. Acute Toxicity in *D. magna*

The acute toxicity tests were conducted in accordance with the ABNT NBR 12713 standard [30], where neonates were exposed to the test substance (dilution medium + leachate extracts or dilution medium + pure nanoparticle powder) for a short period of time (48 ± 1 h), aiming to determine the effective concentration that causes immobility and/or mortality in 50% of the organisms after a period of 48 ± 1 h (EC_50, 48h_). The dilution medium used in the test was the ISO medium specified in ISO 6341 [31]. The tests were conducted with 10 neonates per container in duplicate, exposing 20 organisms per dilution. The dilutions used for the 2 types of leachates (reference concrete and concrete with 9% nano-TiO_2_) were 4.2%, 6.25%, 10%, 20%, 50%, and 100%, in addition to the control, which contained only reconstituted ISO water with no exposure to the toxic agent (leachate extract). For example, the 20% dilution consisted of 20% leachate (10 mL) and 80% ISO dilution medium (40 mL) for a total volume of 50 mL. For the pure nanoparticle powder (TiO_2_), the following concentrations were used, corresponding to the ratio between the nanomaterial and the ISO dilution medium: 0 mg/L (control), 1 mg/L, 300 mg/L, 600 mg/L, 800 mg/L, 1000 mg/L, and 1200 mg/L. All suspensions (nano + ISO dilution medium) were dispersed via ultrasonication (Qsonica Q 500 W) at a power of 115 W for 2 min.

The results were obtained after an interval of 48 ± 1 h, with the counting of immobilized and/or dead neonates at each dilution. The data were evaluated via one-way ANOVA via the statistical software GraphPad Prism^®^ (version 8.4.3), and the concentrations corresponding to the EC_50_ were determined at 48 h for each test. Finally, the results of the suspensions (nano + ISO dilution medium) were classified according to the EU 93/67/EEC standard [36], where an EC_50_ < 1 mg/L indicates that the material is very toxic to aquatic organisms; an EC_50_ between 1 and 10 mg/L indicates toxicity; an EC_50_ between 10 and 100 mg/L indicates that the material is harmful; and an EC_50_ > 100 mg/L indicates that the material is not classified.

#### 3.4.2. Chronic Toxicity in *D. magna*

The chronic toxicity test was conducted according to ISO 10706 [37] and the Organization for Economic Cooperation and Development (OECD) test No. 211 [38], with the aim of evaluating sublethal effects on the organisms. In this test, neonates were exposed for 21 days to toxic agents, a period that comprises a significant part of the organism’s life cycle, and at the end of the period, the effects were verified on the following parameters: longevity (number of survivors), size (length), reproduction (number of neonates generated in each posture), and age of first posture. The dilution medium used in the test was the M4 medium described in ISO 6341 [31]. Notably, in the case of tests with pure powders, all suspensions were dispersed via ultrasonication (Qsonica Q 500 W) at a power of 115 W for 2 min.

Table 4 shows the concentrations used in the chronic toxicity tests. Each concentration, as well as the control, was composed of ten 50 mL beakers containing 1 neonate organism per beaker.

After the tests were completed, the results were inputted into the GraphPad Prism^®^ statistical software, and analysis of variance (ANOVA) was performed to define significant differences between groups through the variability between them. This analysis is based on the principle of comparison between the results obtained and the test control (*p* < 0.05). The lowest concentration at which the significant difference exceeds this value is considered the LOEC (lowest-observed-effect concentration). Once the LOEC was determined, the NOEC (no-observed-effect concentration) was defined as the concentration below the LOEC.

## 4. Results and Discussion

### 4.1. Characterization of the Leachate Extracts

The analysis of liquid samples obtained through the leaching process of the concrete via ICP–MS aimed to verify and quantify the presence of titanium (Ti) in the leachates. The results, based on the average of three determinations, revealed that the leachate from the concrete containing nano-TiO_2_ had 2.60 ± 0.08 µg/L of titanium. However, since ICP-MS does not differentiate between dissolved titanium ions and TiO_2_ nanoparticles or their aggregates, the detected Ti may represent suspended TiO_2_ particles rather than free ions. In contrast, the leachate from the reference concrete did not contain the analyte of interest.

**Figure 5:**
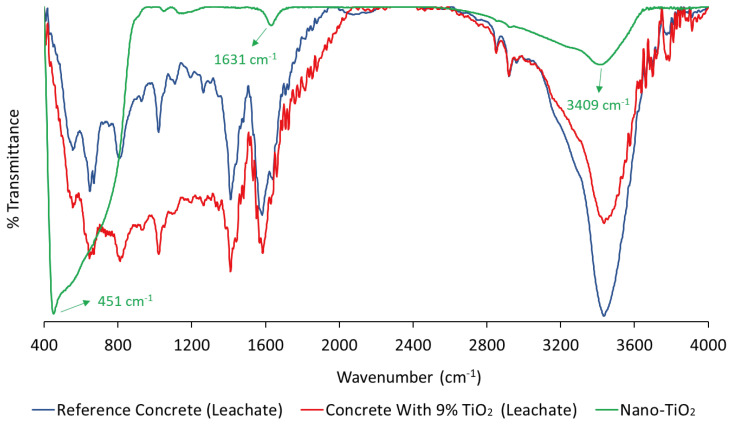
Spectra obtained from the FTIR technique for the leachate samples and for the pure nanoparticle powder of TiO_2_.

Figure 5 presents the FTIR spectra obtained for the leachate extracts of the reference concrete and the concrete with nano-TiO_2_, as well as for the pure nanoparticle powder of TiO_2_.

According to Figure 5, in the spectrum of nano-TiO_2_, it is possible to observe the chemical bond between Ti (titanium) and O (oxygen) atoms, i.e., the Ti–O bending mode and O–Ti–O stretching (anatase) in the region of ≈451 cm^−1^. Additionally, the band at ≈1631 cm^−1^ is characteristic of vibrations of the O-H group from adsorbed water molecules, and the band in the region of ≈3409 cm^−1^ is due to symmetric and asymmetric stretching of hydroxyl groups. Several studies corroborate this interpretation [39,40,41].

A comparison of the FTIR results of the leached extract from the concrete with 9% nano-TiO_2_ with those of the pure powder revealed that the characteristic band of titanium–oxygen bonds in the region of ≈451 cm^−1^ was not present in the spectrum of the leachate. The same observation holds true when comparing the FTIR results of the leached extract from the reference concrete with those of the pure nano-TiO_2_ powder. Furthermore, the spectra of the two leachates are similar. Thus, it was not possible to detect the presence of TiO_2_ nanoparticles in the leached extracts from the concrete via the FTIR technique, indicating that there was no substantial release of the particles, which is characteristic of excellent adhesion of TiO_2_ in the hydration products of the cement. However, the presence of nanoparticles in small quantities, at levels undetectable by the applied technique, cannot be ruled out. Finally, the spectroscopy results are consistent with the findings from the ICP–MS technique, where a very low concentration of titanium ions (2.60 ± 0.08 µg/L) was identified in the leached extract from the concrete with nano-TiO_2_.

The substantial absence of titanium detection in the leachates can be primarily attributed to factors related to the physicochemical behavior of the nanoparticles when incorporated into the cementitious matrix. In this context, anatase has a strong tendency to chemically bond with the hydration products of cement, such as calcium silicate hydrate (C–S–H) [42]. These chemical interactions result in the efficient anchoring of the nanoparticles within the matrix, significantly reducing their mobility and, consequently, their leaching. Additionally, nano-TiO_2_ exhibits low solubility in aqueous media [43].

### 4.2. Acute Toxicity in D. magna

The results obtained from acute toxicity tests (EC_50, 48h_) for the colloidal suspension (pure nanoparticle powder) were extremely high, at 1074.20 mg/L for TiO_2_, and were considered “not classified” under the EU directive 93/67/EEC [36]. These results are consistent with other studies on the acute effects (standard test) of nano-TiO_2_ on *D. magna*, which did not find significant effects: Novak et al. [44] (anatase with an average particle size of 5 nm) and Zhu et al. [45] (anatase/rutile in an 80%/20% ratio with an average particle size of 20 nm).

The high EC_50, 48h_ values for the metal oxide are possibly linked to particle agglomeration, the formation of a monodisperse system, and the instability of the suspension in the ISO medium, which are factors that reduce the degree of toxicity. As shown in Table 2, the formation of agglomerates was confirmed by the effective diameter of the nanomaterials in the ISO medium (acute toxicity), which was 1708.58 ± 6.96 nm. The presence of dissolved salts in the medium alters the surface charge of the particles, facilitating the formation of agglomerates. A polydispersity index of less than 0.3 (0.21 ± 0.03) indicates the formation of monodisperse systems. The zeta potential of the nanoparticles was negative (−2.85 ± 0.09), indicating instability due to the agglomerates in the suspension.

The toxicity of metal oxide nanoparticles, such as TiO_2_ and ZnO, varies widely depending on several factors, including the nature of the nanoparticles, the test medium, experimental conditions, and the organism being tested. ZnO nanoparticles generally exhibit higher toxicity compared to TiO_2_ [46]. The high toxicity of ZnO is associated with its ability to dissolve in water and release Zn^2+^ ions, which are highly toxic to many aquatic organisms, causing oxidative stress and cellular damage.

The lowest EC_50, 48h_ value was for the extract from the concrete with TiO_2_ (64.6 mL/L), followed by the reference extract (104.0 mL/L). The results show that the addition of titanium dioxide nanomaterials to the cementitious matrix reduces the EC_50, 48h_ value of the reference leachate extract; the incorporation of 9% TiO_2_ resulted in a 38% reduction. These results suggest that the incorporation of nanoparticles into the concrete, and consequently, their interaction with Portland cement compounds, produced more complex leachates that are potentially more toxic to the test organism.

This finding is supported by the ICP–MS results, which indicated the presence of titanium in the leachate from the nanomodified matrix. While FTIR did not detect TiO_2_ nanoparticles in the leachate, this does not rule out the possibility that Ti was present, as the FTIR technique may not be sufficiently sensitive to detect small quantities. According to the ICP–MS analysis, the leachate from the concrete with 9% TiO_2_ had a titanium concentration of 2.60 ± 0.08 µg/L, which is likely due to the presence of some TiO_2_ particles. However, it is important to emphasize that this concentration is considered extremely low. In contrast, no titanium was detected in the conventional concrete leachate. Therefore, the presence of titanium detected in the leachate may have contributed to the increased acute toxicity observed.

### 4.3. Chronic Toxicity in D. magna

The results obtained from chronic toxicity tests with the colloidal suspension (pure nanoparticle powder) and the two leachates are presented in Table 5, Table 6 and Table 7. In the chronic toxicity test, the parameters analyzed were longevity, size, reproduction, and age of first posture. The concentrations that had a statistically significant effect (*p* < 0.05) on each of these parameters were classified as LOEC (lowest-observed-effect concentration), and the concentrations tested immediately below were classified as NOEC (no-observed-effect concentration). For the tests in which no significant effect (*p* < 0.05) was observed for the evaluated parameters, “NF” was noted, indicating that the concentration causing toxicity was not found.

According to Table 5, for the colloidal suspension with nano-TiO_2_, a significant toxicological effect on longevity (LOEC of 10 mg/L), size (LOEC of 5 mg/L), and reproduction (LOEC of 10 mg/L) was detected after 21 days of exposure. In this scenario, since the chronic toxicity tests have a photoperiod of 16 h with illumination, the effects caused by nano-TiO_2_ are possibly related to the high photocatalytic activity of this semiconductor, which produces reactive species such as the hydroxyl radical (OH·) in the presence of radiation.

The findings from the chronic toxicity tests of the two leachate samples revealed that the reference concrete sample (Table 6) had a significant toxicological effect on only the size parameter, with a LOEC of 4 mL/L. On the other hand, the leachate from the concrete with 9% nano-TiO_2_ (Table 7) did not have a significant toxicological effect on any of the analyzed parameters. In this context, the results indicate that there was no increase in the chronic toxicity of the leachate from the cementitious matrix when nanoparticles were added at a 9% content. Additionally, excellent anchoring of the nanoparticles in the cement hydration products occurred, as they remained adhered after the leaching process. 

This finding aligns with the FTIR spectra, which did not detect the presence of TiO_2_ nanoparticles in the leachate extract. However, it is important to note that the absence of detection in FTIR does not rule out the presence of nanoparticles in quantities too small to be detected by this technique. The titanium detected via ICP–MS likely represents nanoparticles or small aggregates rather than dissolved ions, and this did not result in an amplified effect on chronic toxicity toward *D. magna* compared with the reference concrete.

## 5. Conclusions and Future Prospects

This study explored the toxicological effects of leachates from photocatalytic concrete blocks containing nano-TiO_2_ on *D. magna*, with a focus on the characterization of the leachates and the nanoparticles involved.

Initially, the characterization of the leachates by ICP–MS revealed the presence of titanium in the extracts from the photocatalytic concrete. Although the detected amount was relatively low, the method employed does not allow for the distinction between titanium present in dissolved ionic form or as very small nanoparticles. The absence of TiO_2_ detection in the FTIR analysis suggests that, if nanoparticle release into the aqueous medium did occur, it happened at levels below the sensitivity of the technique, indicating effective anchoring of the nanoparticles within the cement hydration products. This anchoring behavior can be attributed to the strong chemical interaction between the nanoparticles and the cement matrix, which limits their mobility and leaching.

Regarding the toxicity tests, the results indicated that the addition of nano-TiO_2_ to the concrete resulted in an increase in the acute toxicity of the leachates, as evidenced by the reduction in the EC_50_ value at 48 h. This phenomenon may be attributed to the greater complexity of the leachates from the nanomodified matrix, possibly due to the presence of titanium in the form of nanoparticles or small aggregates, which could have contributed to the increased toxicity observed in the test organisms. However, it is important to highlight that, despite the observed increase in acute toxicity, the concrete with 9% nano-TiO_2_ did not exhibit significant chronic toxicological effects on the evaluated parameters (longevity, size, reproduction, and age of first posture) compared to the reference concrete. This suggests that, despite the increase in acute toxicity, prolonged exposure to these leachates did not result in substantial harm over the life cycle of *D. magna*.

For future toxicology studies involving TiO_2_ nanoparticles, it is strongly recommended to use images obtained through High-Resolution Transmission Electron Microscopy (HRTEM). This technique will enable a detailed analysis of structural characteristics at the atomic level, providing essential information on the morphology, size, and distribution of the nanoparticles, as well as potential interactions with other biological or environmental structures. The application of HRTEM can reveal crucial details about the stability of the nanoparticles and their potential influence on toxicological effects, thereby contributing to a deeper understanding of toxicity mechanisms in various experimental contexts. Additionally, in future research, it would be valuable to broaden the scope of toxicological assessments by incorporating a variety of biological species beyond *D. magna*. This could include organisms such as *Danio rerio* (zebrafish), *Pseudokirchneriella subcapitata* (green algae), *Landoltia punctata* (lemnoideae), and *Vibrio fischeri* (luminescent bacteria), each offering unique insights into the potential environmental impacts of photocatalytic concrete leachates containing nano-TiO_2_. Such an approach would enable a more comprehensive evaluation of the ecotoxicological risks, considering different trophic levels and biological endpoints. By doing so, future studies could provide a deeper understanding of how these materials interact with diverse aquatic organisms, thereby offering more robust data for environmental risk assessments.

## Figures and Tables

**Table 1:** Chemical and physical characteristics of nanoparticles.

Characteristics	Nano-TiO_2_
Purity	>99%
Trace elements	Aluminum (Al) ≤ 17 ppm Magnesium (Mg) ≤ 65 ppm Silicon (Si) ≤ 120 ppm Calcium (Ca) ≤ 75 ppm Sulfur (S) ≤ 130 ppm Niobium (Nb) ≤ 80 ppm
Average particle size	10 nm
Morphology of particles	Ellipsoidal and spherical
Specific surface area	≥60 m^2^/g
Crystallographic structure	Anatase
Bulk density	0.2–0.3 g/cm^3^
Density (at 20 °C)	3.9

**Table 2:** Zeta potential, effective diameter, polydispersity index, and pH of nano-TiO_2_ measured in ultrapure water, ISO, and M4.

Nano	Test	Medium		
		Ultrapure Water	ISO	M4
TiO_2_	Zeta potential (mV)	12.89 ± 0.38	−2.85 ± 0.09	3.84 ± 0.35
	Effective diameter (nm)	580.00 ± 1.39	1708.58 ± 6.96	1539.34 ± 8.12
	Polydispersity index	0.16 ± 0.03	0.21 ± 0.03	0.16 ± 0.01
	pH	7.21	7.90	7.62

**Table 3:** Characteristics of Portland cement.

**Chemical Characteristics**	
Loss on ignition	5.43%
Magnesium oxide	5.76%
Sulfur trioxide	2.79%
Sodium oxide	0.23%
Potassium oxide	1.09%
Insoluble residue	11.2%
**Physical Characteristics**	
Fineness—residue on the sieve—0.075 mm	2.3%
Fineness—residue on the sieve—0.044 mm	11.3%
Specific surface—Blaine	372 m^2^/kg
Specific mass	2.970 g/cm^3^
Autoclave expansion	0.73 mm
Water from the paste with normal consistency	27.9%
Time of setting, not less than	257 min
Time of setting, not more than	335 min
Compressive strength (3 days)	33.9 MPa
Compressive strength (7 days)	39.2 MPa
Compressive strength (28 days)	47.4 MPa

**Table 4:** Concentrations used for chronic toxicity tests.

Concentrations	Nano-TiO_2_ (mg/L)	Reference Concrete (mL/L)	Concrete with 9% TiO_2_ (mL/L)
C0 (control)	0	0	0
C1	0.63	0.25	0.5
C2	1.25	0.5	2.0
C3	2.5	1.0	3.5
C4	5.0 *	2.0	5.0 **
C5	10	4.0	6.5

* Example: 5 mg of nano in 1.0 L of diluent medium (M4). ** Example: 5 mL of sample in 0.995 L of diluent medium (M4).

**Table 5:** Results of chronic toxicity tests with *D. magna*: nano-TiO_2_.

Pure Powders of Nanoparticles: Nano-TiO_2_				
Concentrations (mg/L)	Longevity (%)	Size (Length) ± SD (mm)	Reproduction ± SD (Number of Neonates)	Age of First Posture ± SD (Days)
0 (Control)	100	4.125 ± 0.34	3.392 ± 1.56	11.44 ± 1.01
0.63	70	4.385 ± 0.12	2.408 ± 1.84	12.00 ± 1.76
1.25	90	4.176 ± 0.12	2.919 ± 1.26	12.20 ± 1.98
2.5	90	4.371 ± 0.09	2.704 ± 1.16	11.67 ± 1.12
5.0	100	3.713 ± 0.24 *	2.575 ± 0.63	12.70 ± 1.83
10	40 *	3.624 ± 0.25 *	0.417 ± 0.92 *	14.00 ± 0.00
LOEC (mg/L)	10.0	5.0	10.0	>10.0 (NF)
NOEC (mg/L)	5.0	2.5	5.0	>10.0 (NF)

* Result with a statistically significant effect (*p* < 0.05) on the test organisms.

**Table 6:** Results of chronic toxicity tests with *D. magna*: reference concrete.

Leachate: Reference Concrete				
Concentrations (mL/L)	Longevity (%)	Size (Length) ± SD (mm)	Reproduction ± SD (Number of Neonates)	Age of First Posture ± SD (Days)
0 (Control)	90	2.860 ± 0.13	3.533 ± 2.06	12.88 ± 1.13
0.25	100	2.766 ± 0.11	4.090 ± 1.97	12.70 ± 0.83
0.5	80	2.777 ± 0.13	4.550 ± 1.17	11.38 ± 1.30
1.0	90	2.740 ± 0.13	4.511 ± 1.09	13.00 ± 1.22
2.0	100	2.780 ± 0.09	4.510 ± 0.54	12.70 ± 2.40
4.0	100	2.714 ± 0.11 *	3.560 ± 1.55	12.89 ± 1.16
LOEC (mL/L)	>4.0 (NF)	4.0	>4.0 (NF)	>4.0 (NF)
NOEC (mL/L)	>4.0 (NF)	2.0	>4.0 (NF)	>4.0 (NF)

* Result with a statistically significant effect (*p* < 0.05) on the test organisms.

**Table 7:** Results of chronic toxicity tests with *D. magna*: concrete with 9% TiO_2_.

Leachate: Concrete with 9% TiO_2_				
Concentrations (mL/L)	Longevity (%)	Size (Length) ± SD (mm)	Reproduction ± SD (Number of Neonates)	Age of First Posture ± SD (Days)
0 (Control)	100	2.854 ± 0.07	6.830 ± 0.99	10.40 ± 1.26
0.5	100	2.804 ± 0.08	5.670 ± 2.43	11.00 ± 1.58
2.0	100	2.843 ± 0.11	5.140 ± 0.89	11.00 ± 1.88
3.5	100	2.834 ± 0.06	5.850 ± 1.93	10.70 ± 1.63
5.0	100	2.858 ± 0.09	5.590 ± 0.58	10.30 ± 0.67
6.5	100	2.850 ± 0.05	5.480 ± 1.53	10.50 ± 1.58
LOEC (mL/L)	>6.5 (NF)	>6.5 (NF)	>6.5 (NF)	>6.5 (NF)
NOEC (mL/L)	>6.5 (NF)	>6.5 (NF)	>6.5 (NF)	>6.5 (NF)

## Data Availability

Data are contained within the article.
